# Children's academic attainment is linked to the global organization of the white matter connectome

**DOI:** 10.1111/desc.12662

**Published:** 2018-03-13

**Authors:** Joe Bathelt, Susan E Gathercole, Sally Butterfield, the CALM team, Duncan E Astle

**Affiliations:** ^1^ MRC Cognition and Brain Sciences Unit Cambridge University Cambridge UK

## Abstract

Literacy and numeracy are important skills that are typically learned during childhood, a time that coincides with considerable shifts in large‐scale brain organization. However, most studies emphasize focal brain contributions to literacy and numeracy development by employing case‐control designs and voxel‐by‐voxel statistical comparisons. This approach has been valuable, but may underestimate the contribution of overall brain network organization. The current study includes children (*N* = 133 children; 86 male; mean age = 9.42, *SD* = 1.715; age range = 5.92–13.75y) with a broad range of abilities, and uses whole‐brain structural connectomics based on diffusion‐weighted MRI data. The results indicate that academic attainment is associated with differences in structural brain organization, something not seen when focusing on the integrity of specific regions. Furthermore, simulated disruption of highly‐connected brain regions known as hubs suggests that the role of these regions for maintaining the architecture of the network may be more important than specific aspects of processing. Our findings indicate that distributed brain systems contribute to the etiology of difficulties with academic learning, which cannot be captured using a more traditional voxel‐wise statistical approach.

**RESEARCH HIGHLIGHTS**

- First investigation of structural connectome associations of academic attainment in children.
- First direct comparison of voxel‐wise and connectome associations approaches in a developmental sample.

## 1 INTRODUCTION

The literature on structural brain mechanisms supporting reading and math abilities strongly emphasizes the contribution of particular anatomical substrates (Peterson & Pennington, 2015; Kucian, Kaufmann, & von Aster, 2014). These substrates are largely derived a priori from adult neuropsychological models or from the use of statistical procedures optimized to uncover maximal focal differences in canonical case‐control designs (Carter et al., 2009; Matejko, Price, Mazzocco, & Ansari, 2013; Rollins et al., 2009; Van Beek, Ghesquiere, Lagae, & de Smedt, 2014). While these approaches have been found to be highly sensitive to focal anatomical differences in acquired disorders, their applicability to differences with a developmental origin is less clear (Karmiloff‐Smith, 1998). In contrast to classical adult neuropsychology, in which specific functions are mediated by specific brain modules (Luria, 1966; Wernike, 1874), more recent theoretical approaches suggest that specialization arises from the interaction between brain regions over the course of development (Johnson, 2003; Pascual‐Leone, Amedi, Fregni, & Merabet, 2005). As a result of these interactions, developmental cognitive problems are likely associated with changes in brain organization, as problems cascade through the system or are partially compensated for elsewhere. To date these theoretical approaches have rarely been paired with neuroimaging analysis techniques that would be sensitive to these broader changes in organization over development. The current study takes a new approach that focuses not on focal contributions, but instead on how principles of whole‐brain connectivity might be strongly associated with children's literacy and numeracy abilities.

The approach typically taken to understanding the neural basis of developmental cognition is unlikely to capture these developmental cascades for several reasons. First, the use of voxel‐wise statistical comparisons emphasizes the small number of voxels of overlap across children, but is insensitive to broader differences in brain organization. Second, the use of case‐control designs with the strict selection of cases and controls can give a potentially misleading appearance of the relative purity of any cognitive deficit (e.g., Ranpura et al., 2013). In reality, comorbidity is very high in developmental disorders (Gillberg, 2014; Kovas et al., 2007; Landerl & Moll, 2010; Pennington & Bishop, 2009), and the real‐world presentation of difficulties is often not reflected by the cohorts of children studied. Indeed, we are not aware of any studies that link measures of brain structure to continuous measures of literacy and numeracy performance across the whole brain. In short, the way we have studied the brain correlates of skills like literacy and numeracy makes two implicit assumptions—first, that there will be a voxel overlap across cases corresponding to the best correlate of that skill, and second, that these are isolated skills. But these assumptions are not in keeping with our theoretical understanding of developmental cognitive disorders, so there could be additional value in taking an alternative approach.

Attainment in reading and math has been extensively studied in more traditional neuroimaging studies (Kucian et al., 2014; Peterson & Pennington, 2015). In the case of reading, case‐control comparisons indicated differences in tracts of the language system, including the inferior and superior longitudinal fasciculus/arcuate fasciculus, posterior corpus callosum, and extreme capsule (Carter et al., 2009; Rollins et al., 2009). For math performance, white matter connections of the parietal and frontal lobe have been implicated (Matejko et al., 2013; Van Beek et al., 2014).

Our alternative approach to studying the relationship between white matter organization and academic attainment is more inclusive than previous studies. The current study is based on a large sample of children (*n* = 133) who were referred for problems in attention, learning and/or memory, by educational and clinical professionals working in various specialist children's services. The sample is therefore not already restricted to children who have met narrowly defined criteria, for example, particular cut‐off scores on cognitive assessments. Instead, the sample consists of children who are struggling educationally and display a broad range of abilities from deficits to age‐expected performance. This also includes deficits in both reading and math, which are known to often co‐occur (Kovas et al., 2007; Landerl & Moll, 2010).

A network science approach was adopted to obtain a comprehensive account of the neuroimaging data. In this, brain regions are described as nodes and their connections as edges. Nodes typically correspond to regions of interest (Dell'Acqua & Catani, 2012; Fornito, Zalesky, & Breakspear, 2015). Edges can represent the strength of white matter connectivity based on diffusion‐weighted imaging (Qi, Meesters, Nicolay, ter Haar Romeny, & Ossenblok, 2015). Graph theory provides a mathematical framework for the analysis of the resulting network (Bullmore & Sporns, 2009; Rubinov & Sporns, 2010), which describes organizational principles like connection efficiency and centralized vs. distributed organization (Sporns, 2013). Graph theory analyses produce a set of metrics that characterize network organization. This includes: global efficiency (E_G_)—the inverse of the distance from any node to any other node; and average clustering coefficient (C_G_)—the extent to which node's neighbours are also neighbours. These metrics can be calculated at a whole‐brain level, or at the level of networks' nodes (brain regions). To investigate how the organization of the structural brain network may relate to academic attainment, we constructed a network that represents white matter connections throughout the brain based on diffusion‐weighted MRI data. We then explored how the organization of this network varied according to children's literacy and numeracy abilities, and whether age‐related differences in structural organization mediate the relationship between academic attainment and age.

White matter network development is characterized by increases in global efficiency between preschool years and adulthood (Hagmann et al., 2010; Huang et al., 2013; Wierenga et al., 2015). Structural brain networks in neurodevelopmental conditions including autism, premature birth, hypoxic‐ischemic injury, and attention‐deficit hyperactivity disorder (ADHD) deviate from this organization (Konrad & Eickhoff, 2010; Pandit et al., 2013). A set of highly‐connected nodes known as hubs is thought to be particularly important (van den Heuvel, Kahn, Goni, & Sporns, 2012; van den Heuvel & Sporns, 2011). Neurodevelopmental and neurodegenerative diseases have been found to show disproportionate anatomical abnormalities in hub regions (Crossley et al., 2014), indicating that they may be critical for understanding differences in brain development (Di Martino et al., 2014). So, in our analysis we also investigated whether the connectivity of hub nodes plays a particularly important role in explaining children's performance and mediating age‐related changes, using a simulated attack of different nodes within the connectome.

## 2 PARTICIPANTS AND METHODS

### 2.1 Participants

All children participated in a large‐scale study at the MRC Cognition and Brain Sciences, the Centre for Attention, Learning, and Memory (CALM) research clinic. At the clinic, children were recruited on the basis of ongoing problems in attention, learning, and/or memory in school and were identified by professionals working in schools or specialist children's community services. Families were invited for an assessment that lasted approximately three hours. The assessment included the academic attainment measures reported here. Exclusion criteria for referrals were significant or severe known problems in vision or hearing that were uncorrected, conditions of known genetic origin, and having a native language other than English. This study was approved by the local NHS research ethics committee (Reference: 13/EE/0157). Written parental consent was obtained and children provided verbal assent.

The initial sample included 206 children. Of this sample, children were selected who had good‐quality MRI data (see Supporting Information for quality control), and completed assessments of math and reading. We further restricted the age range to focus on the age range with most available participants (between the 5th and 95th percentile of the whole sample, which left nine children out of the analysis). The final sample consisted of 133 children (86 male, Age: mean = 9.42, *SD* = 1.715, range: 5.92–13.75y). Sample average cognitive scores in the sample were below age expectation with verbal IQ, short‐term memory, and working memory scores about half a standard deviation below the population mean (see Table 1). Performance on an assessment of vocabulary was within the age‐typical range. Parent questionnaires indicated elevated levels of behaviours associated with Attention Deficit Hyperactivity Disorder (ADHD, Conners Parent Rating Form 3rd edition; Conners, 2013) and behaviours associated with deficits in executive function (Behavior Rating Inventory of Executive Function; Gioia, Isquith, Retzlaff, Espy, 2002; see Table 2). As may be expected for children referred for struggling at school, one‐third of children had received a diagnosis through community services (*n* = 42, 31%). The most common was Attention Deficit Hyperactivity Disorder (*n* = 18, 13%), followed by Dyslexia (*n* = 12, 9%), and autism spectrum disorder (*n* = 5, 3%). Seven children had other diagnoses (dyspraxia: *n* = 2, tic disorder: *n* = 2, deficits in attention, motor, and perceptual abilities: *n* = 2, developmental delay: *n* = 1).

**Table 1:** Cognitive performance and behavioural rating scores in the study sample. Cognitive scores are expressed as *z*‐scores relative to the normative sample of the assessment

Measure	Mean	*SE*	Min	Max	*t*	*P*
Matrix Reasoning^1^	−0.60	0.082	−2.50	2.20	−7.38	< .001
PPVT^2^	−0.05	0.103	−4.33	3.00	−0.51	.614
AWMA^3^ Digit Recall	−0.47	0.102	−2.67	3.27	−4.65	< .001
AWMA Dot Matrix	−0.46	0.082	−2.60	2.33	−5.60	< .001
AWMA Backward Digit Recall	−0.57	0.069	−2.40	2.47	−8.29	< .001
AWMA Mr. X	−0.22	0.080	−2.33	2.87	−2.79	.006

*Note*

^1^Wechsler Abbreviated Scale of Intelligence – Second Edition (Wechsler, 2011). ^2^Peabody Picture Vocabulary Test (Dunn & Dunn, 2007). ^3^Automated Working Memory Assessment (AWMA) (Alloway, 2007).

**Table 2:** Behavioural ratings are shown as T‐scores scaled according to the normative sample of the rating scale

Measure	Mean	*SE*	% elevated
Conners^1^ Inattention	80.10	1.063	88.37
Conners Hyperactivity/Impulsivity	72.50	1.483	68.99
Conners Learning Problems	75.95	1.035	86.82
Conners Executive Function	73.64	1.160	81.40
Conners Aggression	61.78	1.485	44.19
Conners Peer Relations	69.60	1.644	58.91
BRIEF^2^ Metacognitive Index	69.74	0.906	83.72
BRIEF Behavior Regulation Index	65.34	1.281	62.02
BRIEF Global Executive Index	69.16	1.027	78.29

*Note*

^1^ *Conners 3rd Edition Parent Short‐Form* (Conners, 2013). ^2^Behavior Rating Inventory of Executive Function (BRIEF) (Gioia et al., 2002).

### 2.2 Assessment of academic attainment

**Figure 1:**
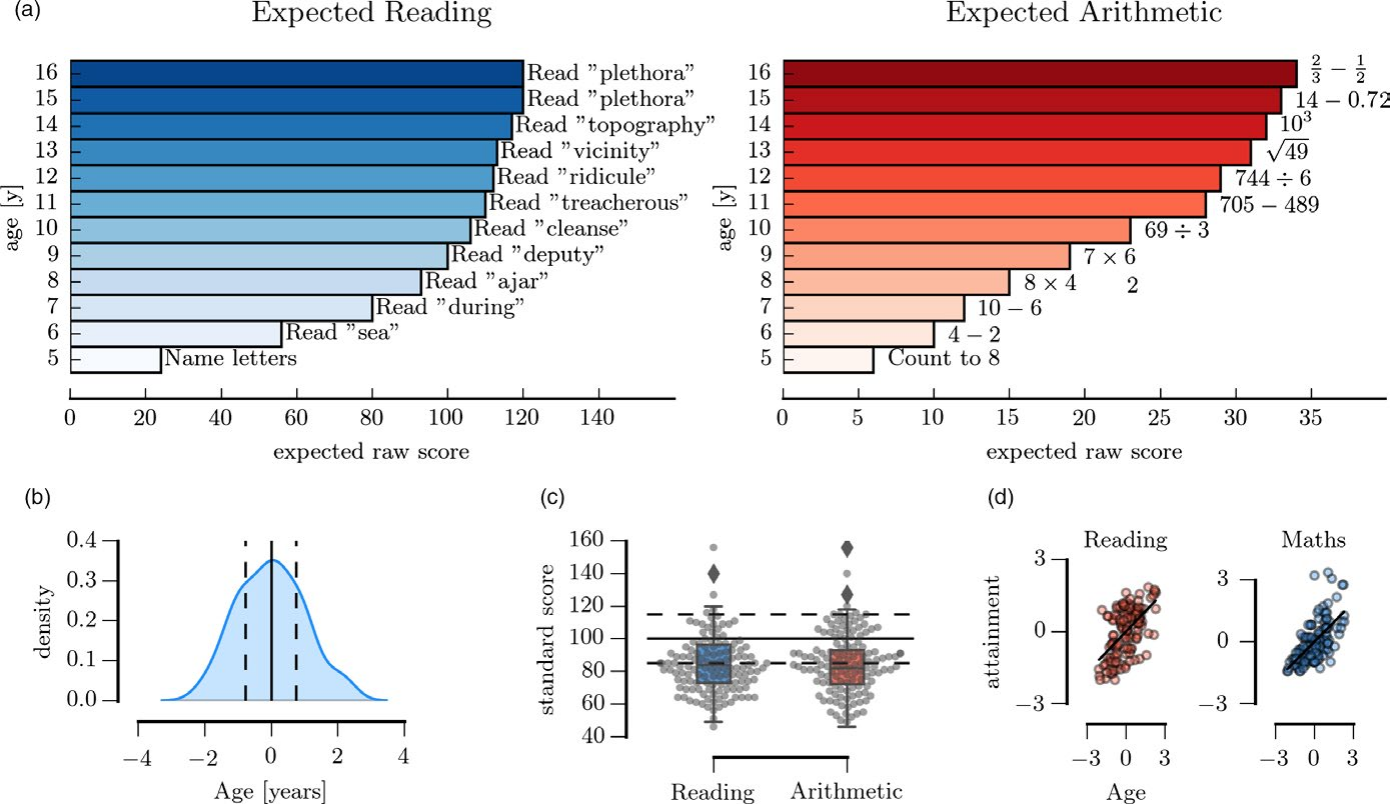
(A) Illustration of skills required to reach age‐appropriate scores at different ages on the Wechsler Individual Achievement Test 2nd edition UK (WIAT‐II UK) Word Reading task (left) and the Numerical Operations task (right). Skills assessed on the attainment measures ranged from basic fact retrieval to complex skills, including the ability to read of non‐phonetic words and solving multi‐step calculations. (B) Age distribution in the current study. The solid line indicates the mean of the sample and the dashed lines show the 25th and 75th percentiles. (C) Distribution of age‐standardized scores for reading and arithmetic in the current sample. The solid line indicates the age‐expected mean and the dashed lines show scores ± 1 standard deviation around the age‐expected mean based on the standardization sample. (D) Relationship between participant age and academic attainment scores. Scores represent raw scores scaled to the mean and standard deviation of the sample (*z*‐transformed)

Children completed the Numerical Operations and Word Reading subset of the Wechsler Individual Achievement Test‐Second UK Edition (WIAT‐IIUK) (Wechsler, 2005) as part of a larger battery of tests in an individual assessment setting with a trained researcher. For the Numerical Operations subtest, children had to solve arithmetic problems in a booklet that ranged from basic counting to more complex operations including multi‐digit multiplication and calculations with fractions. For the Reading subtest, the child had to read single words from a card that got progressively more difficult and correct pronunciation was scored (see Figure 1A). For both tests, correct responses were scored until six consecutive scores of zero were reached following the reference manual of the test (Wechsler, 2005).

### 2.3 MRI data acquisition

Magnetic resonance imaging data was acquired at the MRC Cognition and Brain Sciences Unit, Cambridge, UK. All scans were obtained on the Siemens 3 T Tim Trio system (Siemens Healthcare, Erlangen, Germany), using a 32‐channel quadrature head coil. The imaging protocol consisted of two sequences: T1‐weighted MRI and a diffusion‐weighted sequence.

T1‐weighted volume scans were acquired using a whole‐brain coverage 3D Magnetization Prepared Rapid Acquisition Gradient Echo (MP RAGE) sequence acquired using 1 mm isometric image resolution. Echo time was 2.98 ms, and repetition time was 2250 ms.

Diffusion scans were acquired using echo‐planar diffusion‐weighted images with an isotropic set of 60 non‐collinear directions, using a weighting factor of b = 1000s*mm‐2, interleaved with a T2‐weighted (b = 0) volume. Whole brain coverage was obtained with 60 contiguous axial slices and isometric image resolution of 2 mm. Echo time was 90 ms and repetition time was 8400 ms.

### 2.4 Connectome construction

**Figure 2:**
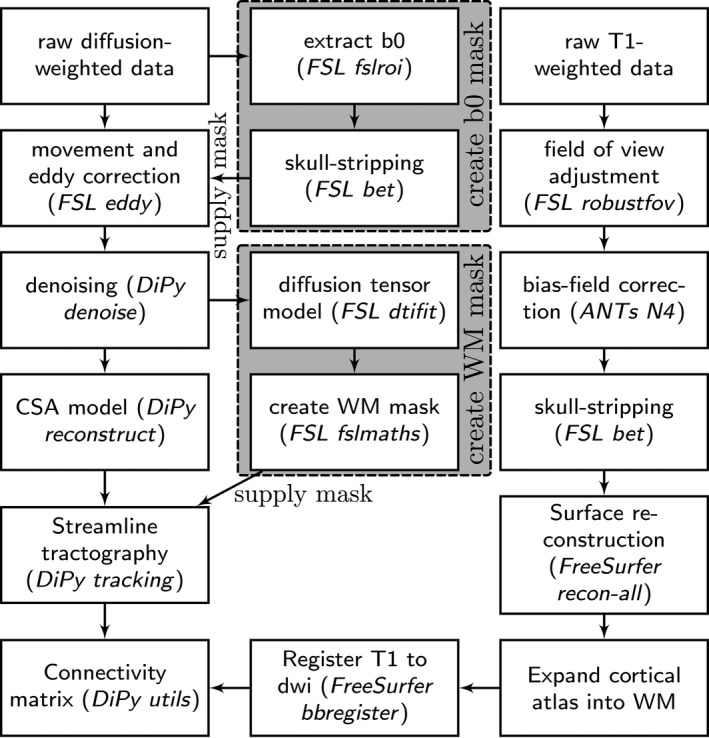
Overview of processing steps used to create white matter connectomes from structural MRI data

The white matter connectome reconstruction followed the general procedure of estimating the most probable white matter connections for each individual, and then obtaining measures of fractional anisotropy (FA) between regions (see Figure 2). The details of the procedure are described in the following paragraphs and followed the same procedure as previously employed (Bathelt, Barnes, Raymond, Baker, & Astle, 2017). In the current study, MRI scans were converted from the native DICOM to compressed NIfTI‐1 format (dcm2nii). Subsequently, a brain mask was derived from the b0‐weighted volume of the diffusion‐weighted sequence and the entire sequence was submitted for correction for participant movement and eddy current distortions through FSL's eddy tool. Next, non‐local means de‐noising (Manjón, Coupé, Martí‐Bonmati, Collins, & Robles, 2009) was applied using the Diffusion Imaging in Python (DiPy) v0.11 package (Garyfallidis et al., 2014) to boost signal‐to‐noise ratio. The diffusion tensor model was fitted to the preprocessed images to derive maps of fractional anisotropy (FA) using dtifit in FSL v.5.0.6 (Behrens et al., 2003). A constant solid angle (CSA) was fitted to the 60‐gradient‐direction diffusion‐weighted images using a maximum harmonic order of 8 using DiPy. Next, probabilistic whole‐brain tractography was performed based on the CSA model with 8 seeds in any voxel with a General FA value higher than 0.1. The step size was set to 0.5 and the maximum number of crossing fibers per voxel to 2.

For ROI definition, T1‐weighted images were preprocessed by adjusting the field of view using FSL's robustfov, non‐local means denoising in DiPy, deriving a robust brain mask using the brain extraction algorithm of the Advanced Normalization Tools (ANTs) v1.9 (Avants et al., 2011), and submitting the images to recon‐all pipeline in FreeSurfer v5.3 (http://surfer.nmr.mgh.harvard.edu). Regions of interests (ROIs) were based on the Desikan‐Killiany parcellation of the MNI template (Desikan et al., 2006) with 34 cortical ROIs per hemisphere and 17 subcortical ROIs (brain stem, and bilateral cerebellum, thalamus, caudate, putamen, pallidum, hippocampus, amygdala, nucleus accumbens). The surface parcellation of the cortex was transformed to a volume using the aparc2aseg tool in FreeSurfer. Next, the parcellation was expanded by 2 mm into the subcortical white matter using in‐house software. In order to move the parcellation into diffusion space, a transformation based on the T1‐weighted volume and the b0‐weighted image of the diffusion sequence was calculated using FreeSurfer's bbregister and applied to the volume parcellation. For each pairwise combination of ROIs, the number of streamlines intersecting both ROIs was estimated and transformed to a density map. A symmetric intersection was used, that is, streamlines starting and ending in each ROI were averaged. The weight of the connection matrices was based on fractional anisotropy (FA). To obtain FA‐weighted matrices, the streamline density maps were binarized after thresholding at five streamlines per voxel and multiplied with the FA map and averaged over voxels to obtain the FA value corresponding to the connection between the ROIs. This procedure was implemented in‐house based on DiPy v0.11 functions (Garyfallidis et al., 2014). In summary, the connectomes presented in the main analysis represent the FA value of white matter connections between cortical and subcortical regions of interest.

### 2.5 Graph theory analysis

Graph metrics of node degree, mean strength, local and average clustering coefficient (C_j_, C_G_), and local and global efficiency (E_j_, E_G_) were calculated using the python implementation of the Brain Connectivity Toolbox as described in Rubinov and Sporns (2010). The influence of mean strength and graph density was removed by normalizing the graph metrics to randomly shuffled networks with the same degree of distribution, density, and mean strength (Fornito, Zalesky, & Bullmore, 2016). Connectivity matrices are typically thresholded to remove spurious connections, but the choice of the inclusion threshold is quite arbitrary. For the current analysis, a range of thresholds was used and the area under the curve was calculated (van Wijk, Stam, & Daffertshofer, 2010).

### 2.6 Statistical analysis of the graph metrics

The relationship between global graph metrics and academic attainment measures was investigated in multiple regression models with the attainment measures as the outcome. Raw scores were used for all the statistical models, rather than using age standardized scores. There are two important reasons for doing this: (i) keeping age separate meant that we could specifically test to the role of age in our model, and use both quadratic and linear terms; and (ii) we could subsequently conduct a mediation analysis using age. Global graph metrics were entered into the model as predictors alongside control variables for brain volume and movement during the diffusion sequence (see below). Model:

$$\begin{array}{cc} Y_{\mathrm{attainment}}\;=\; & \beta_{\mathrm{graphmetric}}X_{\mathrm{graphmetric}}+\beta_{\mathrm{brainvolume}}X_{\mathrm{brainvolume}}+\beta_{\mathrm{movement}}X_{\mathrm{movement}}\\ & +\beta_{\mathrm{intercept}}+\epsilon \end{array}$$

Bonferroni correction was used to adjust for multiple comparisons associated with the two outcomes (reading, maths) and the two predictors (C_G_, E_G_) leading to an adjusted significance criterion of *p* < .0125.

Influence of extraneous variables: We investigated the influence of total brain volume estimated through FSL SIENA (Smith et al., 2002) from T1‐weighted images and movement during the diffusion sequence estimated through FSL eddy (Graham, Drobnjak, & Zhang, 2016). Total brain volume was not related to math or reading performance (Math: *F*(1, 129) = 0.36, β = −0.05, *p* = .550; Reading: *F*(1, 129) = 3.00, β = −0.15, *p* = .086). There was a significant effect of total brain volume on the C_G_ (*F*(1, 129) = 9.97, β = −0.27, *p* = .002) and on E_G_ (*F*(1, 129) = 7.64, β = −0.24, *p* = .007). There was an effect of participant motion on math scores (*F*(1, 129) = 4.78, β = −0.19, *p* = .031), indicating that participants who moved more during the sequences scored lower on the math attainment task. Participant motion was not related to reading attainment, the C_G_, or the E_G_ (reading: *F*(1, 129) = 0.84, β = −0.08, *p* = .360, C_G_: *F*(1, 129) = 0.63, β = 0.07, *p* = .429; E_G_: *F*(1, 129) = 0.16, β = 0.04, *p* = .690). Total brain volume and participant motion were included as control variables in all analyses that used the GLM approach.

Investigation of age effect: The influence of age was investigated by including and excluding age as a control variable with a linear and quadratic term:

$$Y_{\mathrm{attainment}}=\cdots +\beta_{\mathrm{age}}X_{\mathrm{age}}+\beta_{\mathrm{age}}X_{\mathrm{age}}^{2}$$

In addition, we investigated whether age‐related improvements in academic attainment measures are mediated by age‐related differences in structural connectome organization. To this end, *z*‐transformed variables of age, connectome metrics, and attainment measures were entered in mediation models using the lavaan package v0.5‐23.1097 under R 3.4.1. Variables for brain volume and movement were entered as control variables of no interest. Standardized indirect effects were estimated in a bootstrap procedure with 1000 samples (Rosseel, 2012).

Node‐level association between graph metrics and academic attainment: The relationship between node‐level graph metrics of local clustering and local efficiency with reading and math scores was investigated using regression models for each region with control variables for brain volume, movement, and age (linear, squared).

### 2.7 Simulated attack on connectome nodes—the importance of hub nodes

In order to assess the role of highly connected nodes for the relationship between global graph measures and ability scores, we carried out a simulated attack on highly connected nodes (rich club), peripheral nodes, and randomly chosen nodes. To this end, between one and 20 nodes from each category were selected at random and their existing edge weights were knocked‐down to the lowest observed value in the group‐average network, that is, 0.01. Then, C_G_ and E_G_ were calculated in the targeted network. This process was repeated 100 times at each step and the results were averaged to remove effects associated with any particular node. Nodes were not removed completely to keep the number of nodes in the network constant. Different knock‐down values (0.001, 0.0001) produced similar results to the reported findings. Node‐level metrics (C_j_, E_j_) were calculated and averaged for hub nodes or peripheral nodes to investigate their association with academic attainment measures.

### 2.8 Comparison of lower and higher performing groups using voxel‐wise tact‐based spatial statistics

In order to contrast the structural connectome approach with more commonly used voxel‐wise statistical analysis, FA maps were processed using tract‐based spatial statistics (TBSS) as implemented in FSL v5.0.9 (see Smith et al., 2006, for detailed description of TBSS). In short, FA maps were moved to common space via an affine and non‐linear transformation using FSL tools. A common template constructed from a large developmental sample constructed using advanced normalization tools (ANTs) v1.9 (Avants et al., 2011) was used as the registration target in the current sample (see Bathelt et al., 2017). Next, the mean FA image was created and thinned to create a mean FA skeleton which represents the centres of all tracts common to the group. Each subject's aligned FA data were then projected onto this skeleton.

For group comparisons, participants with a deficit in academic attainment measures (age‐standardized attainment score more than 2 standard deviations below the age‐expected mean, i.e., < 70) were compared to participants who scored in the typical range expected for their age (age‐standardized attainment score 90 < score < 120). See Table 3 for descriptive statistics characterizing the groups used for voxel‐wise statistical comparison. The groups were compared in an independent sample *t* test model using a permutation‐based algorithm with cluster‐free threshold enhancement as implemented in FSL randomize (Winkler, Ridgway, Webster, Smith, & Nichols, 2014). It is necessarily the case that whenever you form groups, sample size and mean group difference are traded off against one another. The more extreme the between‐group difference created, the smaller the groups. The Supporting Information includes multiple different analyses showing alternative ways of defining case and control groups. In addition, an analysis with reading and math scores as continuous variables was performed. Separate models were run for positive and negative associations between FA and academic assessment scores. The models controlled for age, brain volume, and movement, and contained an intercept term.

**Table 3:** Characteristics of case‐control groups that were used for voxel‐wise statistical comparison

Group	*N*	Age		Attainment score		Motion		Brain volume	
		mean	*SD*	mean	*SD*	mean	*SD*	mean	*SD*
High reading	47	113.34	21.058	102.19	8.358	1.09	0.512	1.76	0.132
Low reading	27	114.70	17.730	60.48	5.879	1.14	0.541	1.77	0.064
*t*		0.28		−22.86***		0.36		0.53	
High maths	39	108.33	21.043	103.46	12.363	1.00	0.450	1.77	0.141
Low maths	30	117.47	16.506	63.07	5.471	1.17	0.591	1.76	0.070
*t*		1.96		−16.66**		1.35		−0.48	

**p < .01; ***p < .001.

## 3 RESULTS

At the group level, ability scores in math and reading were in the low range (age‐normed standard scores: math: mean = 83.41 *SE* = 1.459, reading: mean = 85.41 *SE* = 1.538, see Figure 1C), with 55% of participants scoring 1 standard deviation or more below the norm mean for math and 50% for reading. Nineteen children had difficulties with math (age‐standardized scores: math < 85, reading > 90), 10 children had difficulties with reading (reading < 85, math > 90), 44 children had difficulties in math and reading (math < 85, reading < 85), and 25 children scored in the typical range for both reading and math (math > 90, reading > 90). These scores indicate that even though children were referred on the basis of struggling at school, their ability level spanned the whole range of the spectrum, including children with age‐appropriate learning skills, more selective deficits in literacy or numeracy, and broader shared impairments.

### 3.1 Small‐world organization

**Figure 3:**
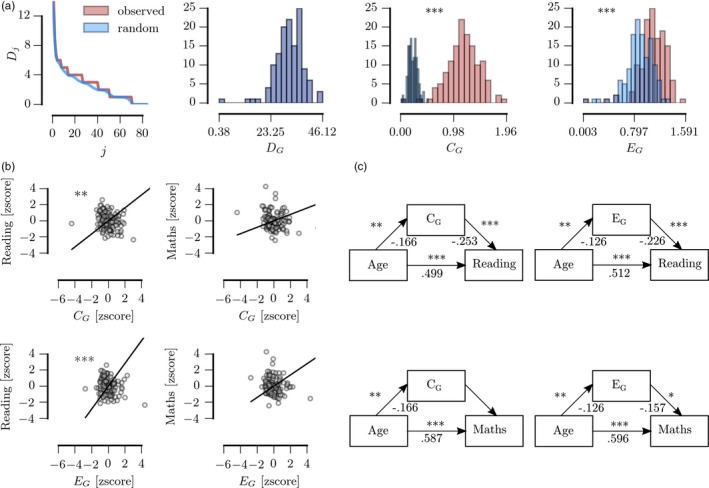
(A) Comparison of the observed structural networks (red) and random networks with the same degree distribution and density (blue) (B) Regression analysis with attainment scores as the outcome and average clustering (C_G_) and global efficiency (E_G_) after regressing the effect of brain volume, movement, and age. (C) Summary of mediation analysis. The values indicate the beta weights of each connection. Legend: ***p < .001; ** *p* < .01; * *p* < .05; B & C: Bonferroni‐corrected *p*‐values are shown

The observed structural networks had higher C_G_ (Observed: mean = 1.16, *SE* = 0.026, Random: mean = 0.23, *SE* = 0.007; paired sample *t* test: *t*(130) = 34.81, *p* < .001) and higher E_G_ (Observed: mean = 1.08, *SE* = 0.019, Random: mean = 0.87, *SE* = 0.018; paired sample *t* test: *t*(130) = 8.12, *p* < .001) compared to scrambled networks with the same degree of distribution and connection strength indicating small‐world organization in the observed structural networks (see Figure 3A).

### 3.2 Relationship between global graph metrics and academic attainment scores

Regression analysis indicated a significant relationship between learning attainment measures and global graph metrics (see Figure 3B, Reading: C_G_: β = 0.37, *p* < .001, C_G_: β = 0.44, *p* < .001; Math: C_G_: β = 0.28, *p* = .007; E_G_: β = 0.32, *p* = .002). The relationship between global graph metrics and reading remained when regressing the linear and quadratic effect of age (see Figure 3C, C_G_: β = 0.24, *p* = .009, E_G_: β = 0.30, *p* < .001) but not for math (C_G_: β = 0.40, *p* = .457; E_G_: β = 0.70, *p* = .189).

Next, a mediation analysis was carried out to investigate the relationship between age, global graph metrics, and academic attainment scores. C_G_ was found to be a significant mediator of age‐related improvements in reading (*F*(3, 131) = 76.653, *p* < .001, mediation effect: *p* = .026, see Figure 3D) but not math (*F*(3, 131) = 74.556, *p* < .001, mediation effect: *p* = .131). Similarly, E_G_ significantly mediated age‐related improvements in reading (*F*(3, 131) = 70.765, *p* < .001, mediation effect: *p* = .012) but not math (*F*(3, 131) = 76.653, *p* < .001, mediation effect: *p* = .076). No significant mediation effects were observed for the squared age term for reading (C_G_: *F*(3, 131) = 4.641, *p* = .2, mediation effect: *p* = .976; E_G_: *F*(3, 131) = 11.411, *p* = .01, mediation effect: *p* = .977) or math (C_G_: *F*(3, 131) = 2.101, *p* = .552, mediation effect: *p* = .976; E_G_: *F*(3, 131) = 5.148, *p* = .161, mediation effect: *p* = .977)

### 3.3 Relationship between regional variation in graph metrics and academic attainment scores

**Figure 4:**
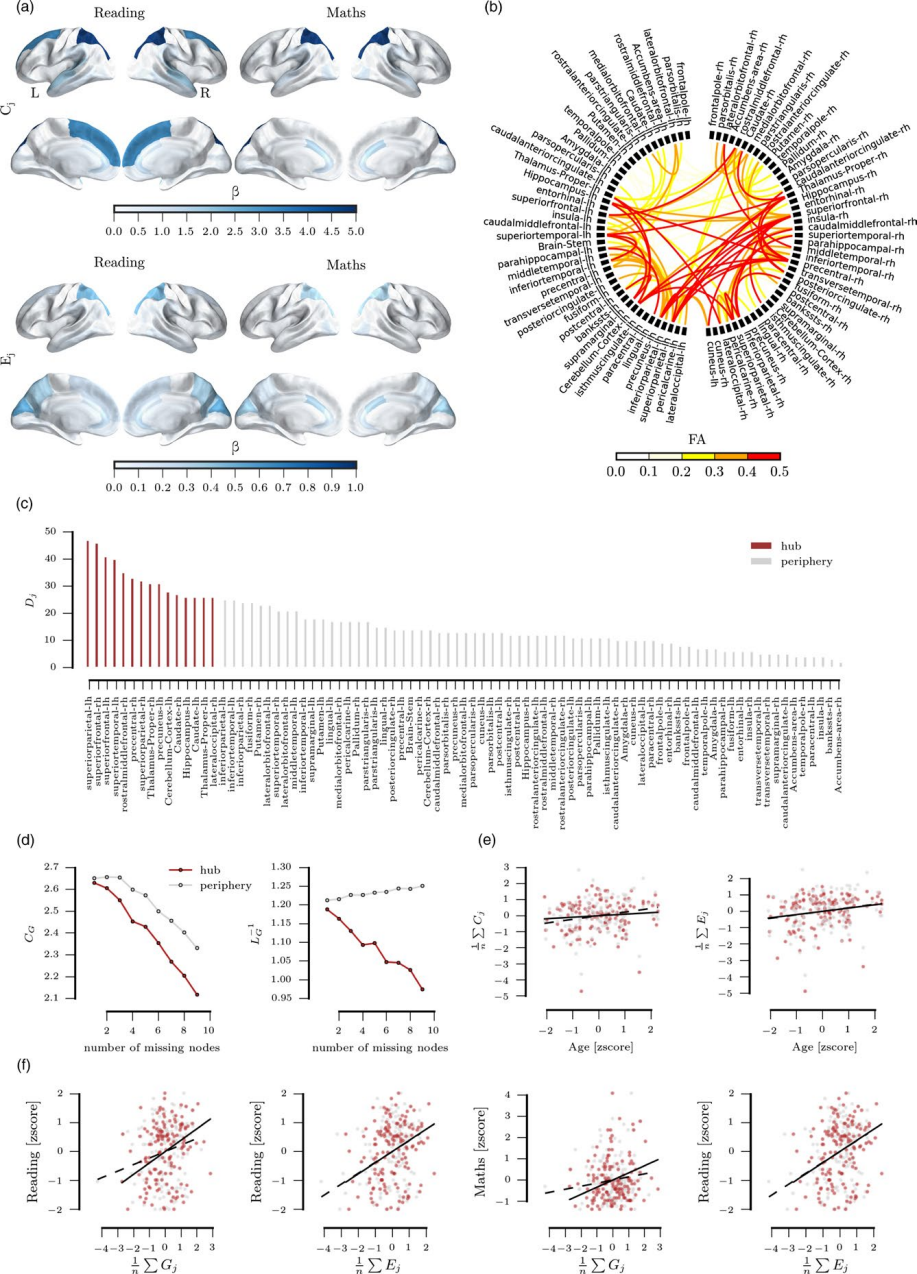
(A)** **Relationship between local clustering (C_j_), local efficiency (E_j_), and maths and reading scores. The colour indicates regression coefficients for each region controlling for the effect of motion, brain volume, and the linear and quadrative effect of age. (B)** **Group‐average connectome thresholded at FA > 0.1 for illustration purposes. (C)** **Degree (D_j_) of nodes in the group‐average connectome. Nodes shown in red are considered hubs with a degree that is one standard deviation above the mean across nodes. (D) C_G_ and E_G_ of the mean network after reducing the connection strength of hub or peripheral nodes. (E)** **Relationship between the average clustering coefficient and average local efficiency of the hub and peripheral nodes with age. The solid line indicates the best fit from the model for hub nodes and the dashed line shows the fit for peripheral nodes. (F) Relationship between graph measures and academic attainment scores. The values represent the residual of the graph measures after regressing the effect of age (linear, squared), brain volume, and movement. The solid line indicates the line of best fit for hub nodes and the dashed line shows the best fit for peripheral nodes one standard deviation above the mean across nodes

Next, the relationship between node‐level graph metrics and academic attainment scores was investigated. Overall, stronger relationships were observed between C_j_ and academic attainment scores than for E_j_. There were also differences between the most closely associated regions between math and reading (see Figure 4A). C_j_ and E_j_ of the right superior frontal cortex (β_C_ = 8.34, β_E_ = 0.48), left precuneus (β_C_ = 6.16, β_E_ = 0.70), and right middle frontal cortex (β_C_ = 3.31, β_E_ = 0.56) was most closely associated with reading scores. Math scores were most closely associated with C_j_ of the right superior frontal cortex (β_C_ = 9.37), the left cerebellum (β_C_ = 2.23), and the left lingual cortex (β_C_ = 2.19), and the E_j_ of the right middle frontal cortex (β_E_ = 0.38), the right superior frontal cortex (β_E_ = 0.34), and the right accumbens area (β_E_ = 0.32), and E_j_ of the right middle frontal cortex (β_E_ = 0.38), the right superior frontal cortex (β_E_ = 0.34), and the right accumbens area (β_E_ = 0.32).

### 3.4 Simulated attack on connectome nodes—the importance of hub nodes

In order to assess the importance of hub and peripheral nodes on brain organization, simulated attacks were carried out. Knock‐down of hub nodes led to a larger decrement in C_G_ and E_G_ compared to peripheral nodes (see Figure 4D). Analysis of the relationship with age indicated significant increases in C_j_ of peripheral nodes (β = 0.27, *p* = .006), but not hub nodes (β = 0.12, *p* = .682, see Figure 4E). E_j_ of hub and peripheral nodes were both positively associated with age (hub: β = 0.23, *p* = .028, periphery: β = 0.26, *p* = .011). Regarding the relationship with academic attainment, reading scores were associated with the C_j_ and E_j_ of hub nodes (C_j_: β = 0.38, *p* < .001, E_j_: β = 0.38, *p* < .001), and, to a lesser extent, E_j_ of peripheral nodes (E_j_: β = 0.34, *p* = .001) but not their C_j_ (C_j_: β = 0.22, *p* = .109). Math was closely related to C_j_ and E_j_ of hub nodes (C_j_: β = 0.31, *p* = .005, E_j_: β = 0.29, *p* = .011), but not to peripheral nodes (C_j_: β = 0.04, *p* > .999, E_j_: β = 0.16, *p* = .560).

### 3.5 Comparison of lower and higher performing groups using voxel‐wise tact‐based spatial statistics

**Figure 5:**
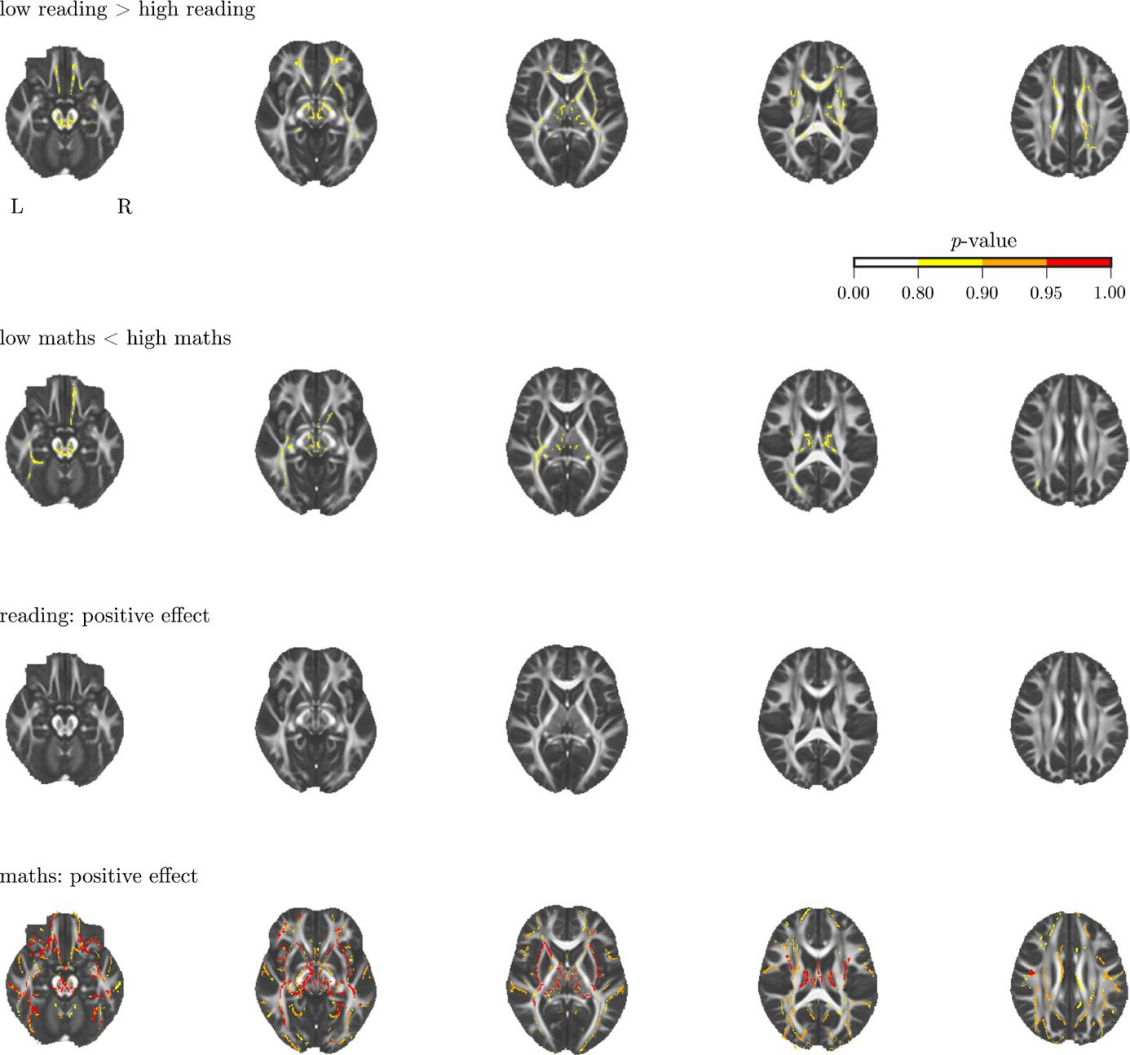
(A) Voxel‐wise comparison of FA values between children with low performance on academic attainment measures compared to children with performance in the age‐expected range. (B) Voxel‐wise analysis of the association between FA values and continuous academic attainment scores. The colours indicate the *p*‐value after correction for multiple comparisons using cluster‐free threshold enhancement with permutation testing

As a contrast with the connectome approach, the data of the current study were also analysed using the more conventional method of voxel‐wise comparisons of children with low performance and those with scores in the age‐expected range (see Table 3 for characteristics of these groups). Voxel‐wise comparison of FA values between the groups with a commonly used method in diffusion imaging, that is, tract‐based spatial statistics, did not indicate significant differences between the groups at *p*_corrected_ < .05 (see Figure 5). As noted in the Methods, this comparison will necessarily include fewer children in order to maximize the group difference (as is typically the case when this approach is taken). However, the Supporting Information includes the results of multiple different partitions of the data, including a median split. None of these result in any significant group differences. When treating literacy and numeracy scores as continuous variables, there was a positive association between higher FA and math scores across with 15% of voxels in the white matter skeleton showing a significant effect at *p*_corrected_ < .05 (see Figure 5B). Voxels showing a positive association with math scores fell into five clusters (see Table 4 for cluster statistics and associated anatomical structures). There were no significant negative associations between FA and math scores, and no significant positive or negative associations for reading scores.

**Table 4:** Characteristics of voxel clusters that showed a significant positive association with math scores in the TBSS analysis

Cluster	Voxels (total/% mask)	*p*_corrected_	MNI coordinates	JHU tracts
1	19172 (14.31)	0.033	10, −29, −18	anterior thalamic radiations (L/R), corticospintal tract (L/R), cingulum (L/R), forceps major, forceps minor, inferior fronto‐occipital fasciculus (L/R), inferior longitudinal fasciculus (L/R), uncinated fasciculus (L/R), superior longitudinal fasciculus (L/R)
2	586 (0.437)	0.049	22, −59, −34	anterior thalamic radiations (L/R), corticospinal tract (L)
3	572 (0.427)	0.049	39, −51, −37	anterior thalamic radiation (L), corticospinal tract (L), inferior fronto‐occipital fasciculus (L), inferior longitudinal fasciculus (L)
4	450 (0.336)	0.046	28, −51, −31	corticospinal tract (L), inferior longitudinal fasciculus (L)
5	155 (0.116)	0.048	−13, −81, 1	cingulum (R), forceps major, inferior longitudinal fasciculus (R), inferior longitudinal fasciculus (R)

*Note*

The second column shows the total number of voxels in the cluster and the proportion relative to number of voxels in the white matter skeleton mask. The fourth column shows the MNI coordinates of the cluster's centre of gravity. The last column shows the labels of white matter tracts in the Johns‐Hopkins University white matter tractography atlas (Hua et al., 2008) that are contained in each cluster.

## 4 DISCUSSION

The current study investigated the relationship between white matter organization and academic attainment in children. We included a large sample of children with performance level spanning low to age‐expected performance, including children with difficulties in reading and math. For the first time this was paired with an analysis method sensitive to organizational principles of the white matter network across the brain. In contrast to previous studies that emphasize focal differences in fronto‐temporal and fronto‐parietal regions (Carter et al., 2009; Kucian et al., 2014; Matejko et al., 2013; Rollins et al., 2009; Van Beek et al., 2014), scores on academic attainment measures were strongly associated with the global architecture of the brain's white matter. A simulated knock‐down of nodes within the connectome showed that this relationship was most strongly related to highly connected hub nodes important for maintaining an optimal architecture. These were more closely associated with reading and math performance than less connected nodes.

The broad association between brain organization and literacy and numeracy stands in apparent contrast to previously published findings that report focal differences in groups with learning deficits. The reason for this apparent disparity may stem from the complementary approaches that are based on different underlying assumptions: case‐control designs focus on selective deficits tightly matched for other cognitive and environmental differences. This can give the impression of relative purity of problems, and standard voxel‐wise statistical approaches tend to emphasize the restricted overlap across cases. Using this approach to compare typical and deficit groups in the current dataset did not indicate any difference in white matter organization for either literacy or numeracy. In contrast, the current study indicates that the overall organization of the brain's white matter plays an important role in academic attainment and that particular regions are important because of their role in maintaining the architecture of the network rather than because of their specific contribution per se. This finding may indicate that broader brain and cognitive systems commonly contribute to the etiology of difficulties with academic attainment, which cannot be captured using a more traditional case‐control approach with voxel‐wise statistical comparisons.

### 4.1 The structural connectome shows small‐world organization

A network science approach also allows us to test how differences across individual tracts contribute to broader differences in brain organization and efficiency. Small‐world organization with high local connectivity and some long‐range connection is thought to be central for optimal information transfer and minimal wiring cost (Bullmore & Sporns, 2009, 2012; Watts & Strogatz, 1998). This organization is present from early in development and can be detected throughout the lifespan (Collin & van den Heuvel, 2013; Dennis et al., 2013; Huang et al., 2013; Tymofiyeva et al., 2013; Vertes & Bullmore, 2014). Small‐world organization can be characterized through graph measures. Higher efficiency indicates that shorter paths throughout the network (Fornito et al., 2015), that is, transfer through the network is faster, more direct, and less prone to noise interference (Bullmore & Sporns, 2009). An increase in global efficiency which is inversely related to characteristic path length (Rubinov & Sporns, 2010), as observed in the current study has been consistently reported in developmental studies (Dennis et al., 2013; Hagmann et al., 2010; Huang et al., 2013; Wierenga et al., 2015). Individual differences in characteristic path length in adults have been found to be highly heritable (Bohlken et al., 2014) and relate to cognitive abilities (van den Heuvel et al., 2009; Koenis et al., 2015). Age‐related decreases in characteristic path length in the current study were found to be predictive of literacy and numeracy scores. This may indicate that structural brain network changes with age relate to improvements in literacy and numeracy as children grow up and progress through school.

Another commonly used measure to characterize brain network organization is the clustering coefficient, which is linked to network segregation. It quantifies the degree of local connectivity between neighbouring nodes. Reports about age‐related changes in the clustering coefficient are mixed with some studies reporting decreases in clustering (Dennis et al., 2013; Hagmann et al., 2010; Tymofiyeva et al., 2013), while other studies find increases (Huang et al., 2013; Wierenga et al., 2015). Discrepancies may be explained by methodological differences between the studies, specifically the metric used to express connection strength. Studies with streamline counts or weighted streamline counts found decreases of the clustering coefficient with age, while studies with diffusion metric‐weighted networks like the current study report increased clustering. Streamline measures may be more influenced by reduced connection likelihood with increasing distance as the brain grows. In contrast, networks based on diffusion metrics follow the developmental tendency of increasing fractional anisotropy with age (Imperati et al., 2011; Westlye et al., 2009), that is, increasing weight in the connectivity matrix leads to a higher global clustering coefficient. The findings of the current study suggest that higher connectivity between neighbouring nodes with age relates to improvements in literacy and numeracy.

### 4.2 The importance of hub nodes

Optimal organization in brain networks has been found to depend on the presence of a small number of highly connected hub nodes (van den Heuvel et al., 2012). These hubs are thought to be very important for the computational capacity of the network (Senden, Deco, de Reus, Goebel, & van den Heuvel, 2014) and are implicated across a range of adult and developmental disorders (Crossley et al., 2014). The hub‐periphery organization is already laid down in the prenatal brain (Ball et al., 2014) and persists across childhood (Grayson et al., 2014) and adolescence into adulthood (Baker et al., 2015). Node‐level analysis implied that hub regions were more closely associated with academic attainment outcomes. Furthermore, a simulated knock‐down of hub nodes had a higher impact on the clustering coefficient and global efficiency compared to attacks on peripheral nodes. Since the global clustering coefficient and global efficiency were found to be predictive of performance on academic attainment tasks, we conclude that hub nodes are central for establishing a network architecture that can support academic attainment optimally.

The association between literacy, numeracy, and brain organization mirrors reported associations between cognitive functions and optimal network structure. For instance, van den Heuvel and colleagues (2009) found that shorter characteristic path length was associated with higher scores on an intelligence scale. This may indicate that optimal network architecture supports both better general cognitive function and higher capacity to learn. Alternatively, optimal network architecture may support particular cognitive abilities that are strongly linked to performance across a range of tasks, for example, executive functions. Such an intermediate level of explanation could provide a fruitful avenue for future investigations.

The association between longer path length, reduced influence of hub nodes, and lower literacy and numeracy scores also resembles findings of atypical brain network organization following early insults like preterm birth, hypoxia‐ischaemia, and intra‐uterine growth restriction (Batalle et al., 2012; Pandit et al., 2013). Further, similar alterations in network organization were also linked to common genetic variants associated with neurodevelopmental disorders like autism and ADHD (Dennis et al., 2013; Hong et al., 2014). This may suggest that various developmental constraints converge on sub‐optimal brain network organization via various mechanisms and that these differences in brain network organization manifest in cognitive and behavioural symptoms commonly observed in developmental disorders.

### 4.3 Comparison of lower and higher performing groups using voxel‐wise tact‐based spatial statistics

The approach taken to understand the structural brain correlates of individual differences in literacy and numeracy in childhood typically makes two implicit assumptions. First, that children can be grouped into those with age‐appropriate skills and those with deficits, and that differences associated with the skill of interest will be highlighted by contrasting the two groups. Second, that children who struggle in a particular domain will have highly overlapping regions of deficit, and can be identified by methods reliant on voxel overlap across cases. We included a canonical analysis using TBSS, which is based upon these two assumptions. It is unable to identify group‐level differences, despite a 2 *SD* difference in educational attainment. One possibility is that this is because assigning groups to maximize between‐group differences reduces the number of children in the analysis. However, the Supporting Material includes various partitions of the data, including a median split. No matter how you carve the data, the TBSS analysis does not identify the group differences. We think there are two important reasons for this. First, the reality is that literacy and numeracy are continuous dimensions of performance, not either “age‐appropriate” or “deficit range”. Any analysis that is sensitive to these continuities will have greater statistical power to identify neural correlates of those effects. This is supported by the wide‐ranging association between FA and math scores that emerged when treating math scores as a continuous variable. Second, the neural effects associated with differences in reading or math need not overlap across children. For example, decreased diffusion within multiple different tracts could result in a similar regional decrease in efficiency, even though there is minimal overlap across children at a voxel level. Even when considering literacy or numeracy as a continuous factor, a voxel‐wise approach would not be able to detect these kinds of effects.

The results of the current study come with some limitations. First, the analyses were based on a sample of children referred for difficulties in school, and academic attainment scores ranged from below‐average to age‐appropriate performance. It is unclear from the current analysis whether the described association between brain organization and academic attainment extends to children with better performance. Another potential limitation concerns the methodology of the connectome construction in the current study. A multitude of methods for constructing structural connectomes from diffusion‐weighted data have been proposed with little validation of methods through histological comparisons (Qi et al., 2015). The methods employed in the current study were chosen to reflect recommended practices (Craddock et al., 2013) and to incorporate FA as a metrics for direct comparison with voxel‐wise analyses, but their relationship to histological measurements remains to be validated.

In conclusion, previous studies on structural brain correlates of literacy and numeracy development suggested the involvement of a limited set of regions and their connections that are specifically linked to aspects of task‐relevant processing. Using a complementary approach that included a range of literacy and numeracy abilities and applied a network‐analytic approach, the current study found that global organization of the white matter network contributes to literacy and numeracy improvements with age. These results suggest that large‐scale neural systems and their interaction play a role in literacy and numeracy development in childhood.

## 5 AUTHOR NOTE

The Centre for Attention Learning and Memory (CALM) research clinic is based at and supported by funding from the MRC Cognition and Brain Sciences Unit, University of Cambridge. The Principal Investigators are Joni Holmes (Head of CALM), Susan Gathercole (Chair of CALM Management Committee), Duncan Astle, Tom Manly and Rogier Kievit. Data collection is assisted by a team of researchers and PhD students at the CBSU. This currently includes: Sarah Bishop, Annie Bryant, Sally Butterfield, Fanchea Daily, Laura Forde, Erin Hawkins, Sinead O'Brien, Cliodhna O'Leary, Joseph Rennie, and Mengya Zhang. The authors wish to thank the many professionals working in children's services in the South‐East and East of England for their support, and the children and their families for giving up their time to visit the clinic.
